# Physical constraints on sand crab burrows: Mechanical properties of wet sand explain the size and spatial distributions of burrows on beaches

**DOI:** 10.1371/journal.pone.0215743

**Published:** 2019-05-01

**Authors:** Ayuko Shinoda, Shin-ichi Fujiwara, Hirofumi Niiya, Hiroaki Katsuragi

**Affiliations:** 1 Department of Earth and Environmental Sciences, Nagoya University, Nagoya, Japan; 2 Museum, Nagoya University, Nagoya, Japan; 3 Center for Transdisciplinary Research, Niigata University, Niigata, Japan; University of California, UNITED STATES

## Abstract

The diameter and vertical depth of sand crab tunnels in sandy beaches are usually restricted to a few centimeters scale and several tens of centimeters, respectively. We designed a study to determine what physical factors restrict tunnel diameter and predict the maximum attainable tunnel diameter and depth. We collected field data on the size and spatial distributions of ghost crab (*Ocypode* spp.) burrows on two sandy beaches (Kawage Beach in Tsu, Mie Prefecture, Japan and Sakieda Beach in Ishigaki, Okinawa Prefecture, Japan), where *O. ceratophthalma* dominants the ghost crab fauna. We measured burrow depths and distance from shoreline in concert with water content of sandy beaches. To explain our observed distributions of crab burrows in the field, we performed experiments in a lab microcosm, comprising a horizontal tunnel through wet sand. We measured the static stability of tunnel structures in relation to water content and two strengths computed from loading force exerted on the sand overlying the tunnels. By comparing field and experimental data, we found that crabs construct their burrows in appropriately wet zones (wet enough to provide sufficient cohesion of the sand grains in tunnel walls to prevent collapse) and that tunnel diameters and depths are sufficiently small to prevent deformation and collapse of their tunnels.

## Introduction

Mechanical properties of tunnels in soil have long been studied by civil engineers [1–5]. The focus of such studies has been to develop safe methods for reinforcing and stabilizing tunnels to prevent them from collapsing. The collapse of voids in cohesive granular matter is aso thought to be responsible for pit structures on comet surfaces [6, 7]. Both the minimum pit size on comets and the maximum size of crab burrows on sandy beaches have been estimated by determining the void stability of a cohesive granular layer [8].

**Fig 1:**
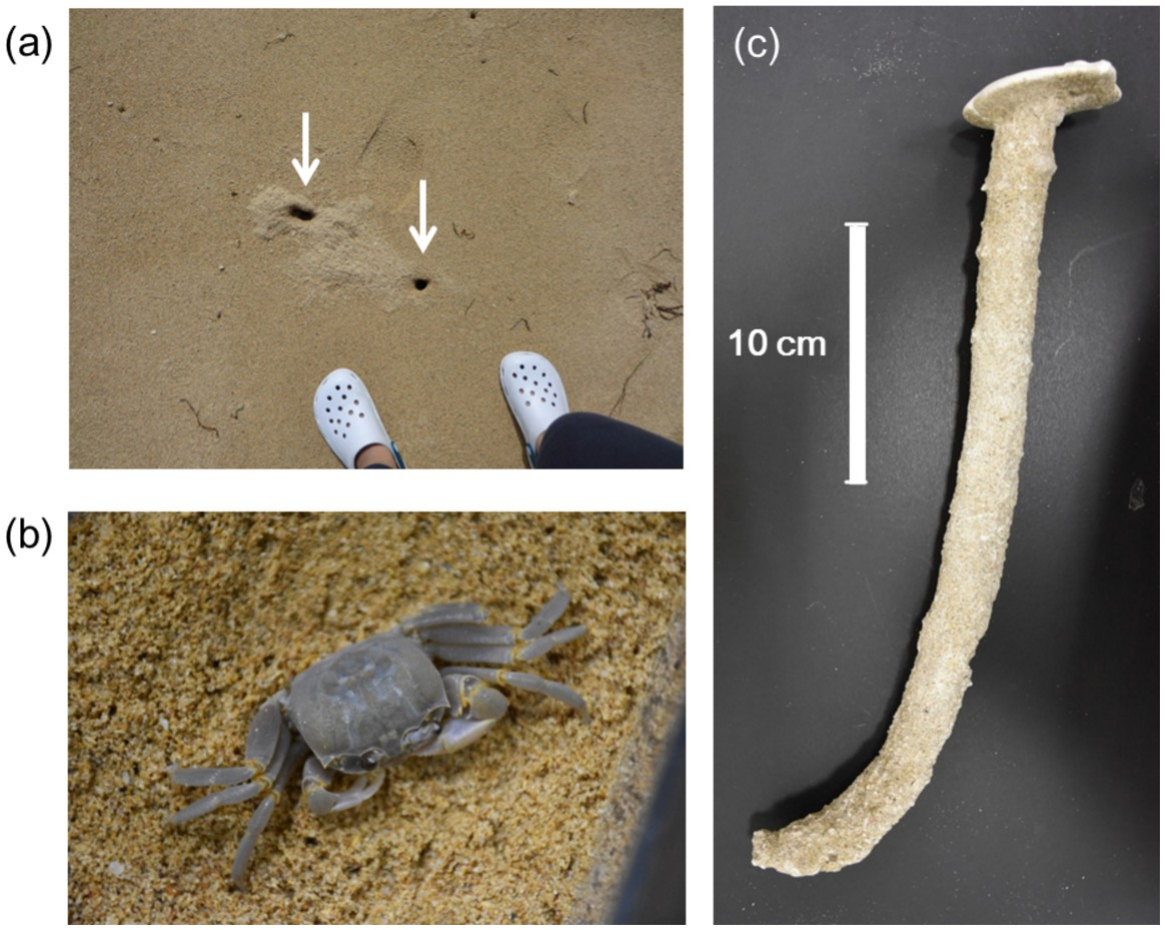
Ghost crabs and their burrows. Panels: (a) Burrow entrances of ghost crabs on a sandy beach on Ishigaki Island, Okinawa Prefecture, Japan (arrows point to the burrow entrances), (b) the horn-eyed ghost crab *Ocypode ceratophthalma*, and (c) a plaster mold (cast) of a ghost crab burrow obtained from in Ishigaki Island, where *Ocypode ceratophthalma* dominants the ghost crab fauna.

*Ocypode* spp. (hereafter we use the word “ghost crabs”) create burrows (Fig 1(a)) on sandy beaches worldwide. In Japan, *O. ceratophthalma* (horn-eyed ghost crab: Fig 1(b)) inhabit sandy beaches from Honshu (main island of Japan) southward to the Yaeyama Islands [9]. *O. ceratophthalma* crabs are nocturnal; they hide in burrows during the day to escape physical stresses (e.g., high temperature) and predators (such as shorebirds) but scavenge and hunt on beaches at night [9]. The entrances of ghost crab burrows are steep, but they have an almost horizontal room at the bottom end of the tunnel (Fig 1(c)). A typical ghost crab burrow is a few centimeters in diameter and several tens of centimeters in depth.

Previous studies have investigated crab burrows from a variety of ecological perspectives. For example, burrowing activities have been shown to support the growth of microorganisms in substrates by increasing drainage and oxidation [10, 11]. Depth of burrows have been used to estimate groundwater level, temperature, moisture, and frequency of crabs emerging during periods of daylight [10–12]. Burrowing activities are also affected by the composition of sands on beaches. For instance, the wall of crab tunnels constructed in loose materials could be reinforced by compressing sand on the walls of the burrows [13]. In addition, fossil crab burrows could provide valuable paleo-environmental information on ancient ecosystems [13].

Crabs build stable burrows in a loose sandy substrate without any engineering knowledge. Perhaps, useful clues for stabilizing tunnel structures can be obtained by an in depth study of crab burrows in the field. To improve our understanding of the mechanical structure of crab burrows, herein we regard the crab burrows as a tunnel structure constructed in a wet granular material (wet sand). By doing so, the mechanical properties of wet granular matter can be used to explain the physical form of crab burrows. The mechanical properties of wet granular matter exhibit various complex behaviors [14–16]. For example, the tensile strength of wet granular matter is dependent on liquid content in a nonlinear manner [17–19]. Complicated behaviors of wet granular matter presumably stem from variations in morphology exhibited by liquids distributed within the granular materials [18, 19]. Wetness regimes can be qualitatively classified by the manner in which liquids are distributed in the granular matter [15]. In fact, the mechanical properties of wet granular matter have often been characterized by using a wetness-regime classification system. In addition, the mechanical properties of wet granular matter depend on packing fraction (bulk density) and grain size [20, 21]. Although there have been studies on the mechanical characterization of wet granular matter, little is known about the stability and/or strength of the tunnel structures formed in a wet granular layer. Only our previous works have discussed the relationship between crab burrows and mechanical structure of wet granular matter [8, 22]. However, detailed comparisons between experiments and actual crab burrows has not been attempted.

The purpose of this research is to gain a better understanding of the relationship between the size and spatial distribution of ghost crab burrows and the environmental conditions of the sand in which the tunnels are constructed. While previous studies have elucidated specific aspects of ghost crabs ecology, most works have been qualitative in nature. In this study, we quantitatively characterize crab burrow structures relative to their mechanical strengths. First, we conducted fieldwork to obtain data on typical burrow size (diameter and vertical depth) and the physical parameters (grain size, water content, and packing fraction) of the sandy substrates in which the burrows occur. Then, we conducted two types of experiments to investigate the mechanical properties of tunnel structures to mimic the physical characteristics of substrate surrounding crab burrows on sandy beaches. In the first experiment, we investigated the range in water content of sands (wet granular substrates) in which a tunnel structure could be maintained. In the second experiment, we examined the strengths of various tunnel structures by loading a statically-stable tunnel structure. As shown in Fig 1(c), a typical crab burrow is almost horizontal at the distal (deepest) end of its tunnel [23]. Therefore, in this study, we applied a quasi-static loading to a horizontal tunnel at a constant speed to examine the stability of the horizontal portion of the burrow. We then discuss the physical constraints on crab-burrow size based on our fieldwork and experimental results. To the best of our knowledge, this study is the first to discuss the relationship between the mechanics of wet granular matter and actual sand crab burrow structures.

## Field-work methods

### Localities

**Fig 2:**
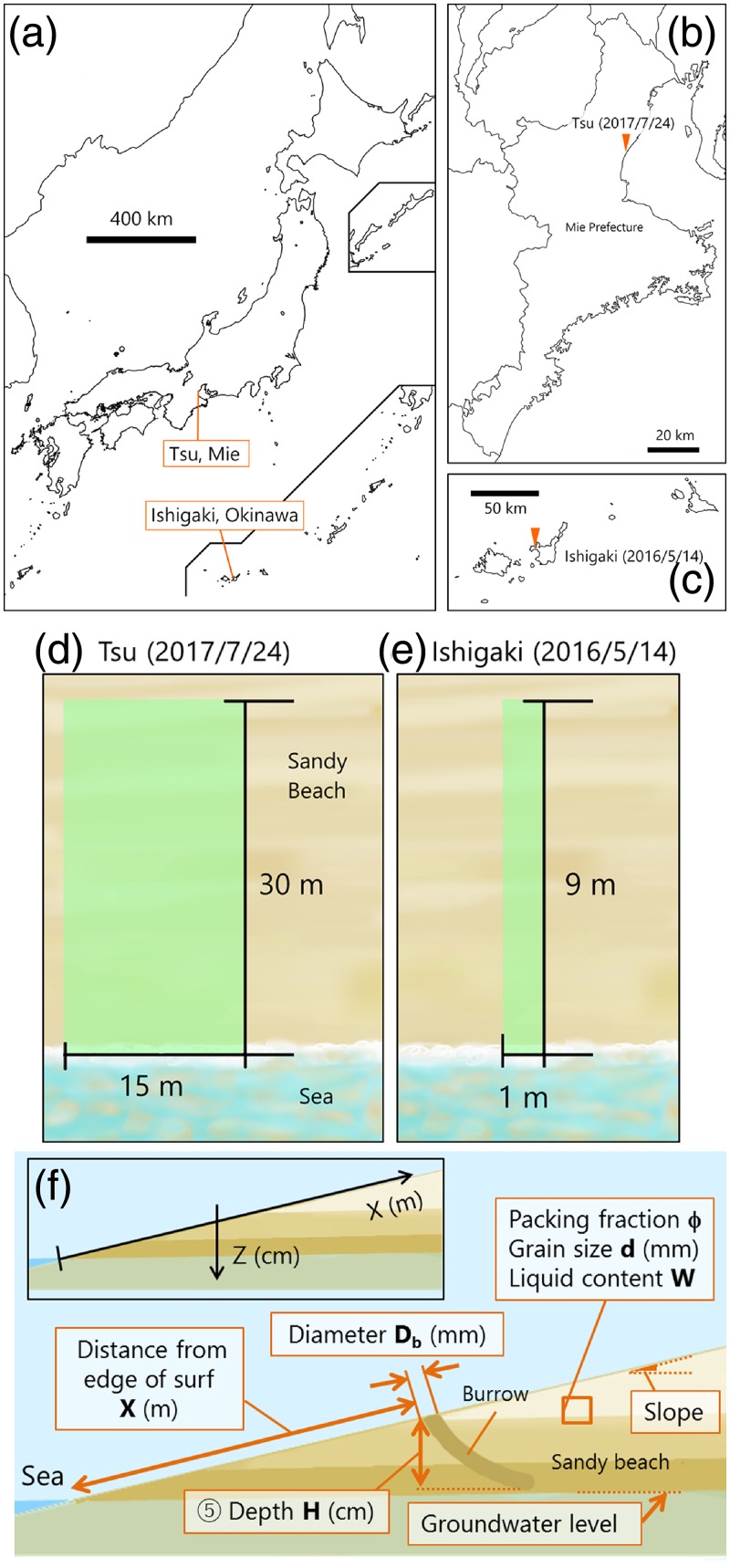
(a) Map of Japan with locations of Tsu, Mie Prefecture, Japan and Ishigaki, Okinawa Prefecture, Japan. Inset maps (b) of Tsu (Kawage Beach) and (c) Ishigaki (Sakieda Beach). Survey area dimensions at (d) Tsu and (e) Ishigaki. For Panel (f), we used the measured quantities and *X*-*Z* coordinate system (inset), where *X* denotes the slope distance from shoreline (along the surface of the beach). *Z* denotes the vertical distance (depth) from the surface to a given point at depth.

Fieldwork was conducted on Kawage Beach in Tsu, Mie Prefecture, Japan (Fig 2(a) and 2(b)), on July 24, 2017 and at Sakieda Beach in Ishigaki, Okinawa Prefecture, Japan (Fig 2(a) and 2(c)) on May 14, 2016. The survey at the Sakieda Beach was permitted by the Yaeyama Promotion Center for Agriculture, Forestry, and Fisheries. Sand samples for our experiments were collected from both beaches, but our field survey of ghost crab burrows was mainly conducted on Kawage Beach in Tsu (i.e., only a limited burrow survey was conducted on Sakieda Beach in Ishigaki). We only measured the relationship between burrow position and water content on Sakieda Beach. All other characterizations of sand were based on our survey results from Kawage Beach. The climate of Kawage Beach is extratropical, whereas Sakieda Beach (located at southwest end of Japan) is subtropical. Kawage Beach was about 350 m long and about 40 m wide when we sampled it, whereas the portion of Sakieda Beach we studied (on the western coast of Ishigaki Island) was about 400 m long and 10 m wide. To minimize the effects in tidal excursions, we performed all field sampling within 2 h windows. We investigated a rectangular area at both beaches (Fig 2(d)–2(f)). A wider beach area was sampled at Kawage Beach, partially because this beach was wider and to obtain more burrow data. Kawage and Sakieda beaches are typical of sandy beaches in Japan in that they harbor an abundance of crab burrows. During our survey periods, mean air temperature at Sakieda Beach was 29°C, and humidity ranged from 74% to 86%, whereas at Kawage Beach, the mean daily temperature range was 30°C–31°C and humidity ranged from 63% to 73%. There had been no rain for more than three days before the surveys (including the day of surveys) at either study site. Therefore, the effect of the rainfall on the water content of sands on the studied beaches can be regarded as negligible.

### Burrow characterization

We mainly investigated the size and spatial distributions of burrows surveyed on July 24, 2017 at the Tsu site (Fig 2(a), 2(b) and 2(d)) (*n* = 54 tunnels). We measured the diameter of burrow entrances (*D*_b_) with a Vernier caliper. The Burrow length (*H*) was measured using a string with a sinker. Because tunnel gradient ranged from 55° to 85° in our observations, the vertical depth of the burrow was about 0.84–0.99 of the value of *H*. Because this factor is close to unity, we used *H* as an approximation for the vertical depth of burrows. In addition, we measured the distribution of burrows on the beach surface using photographs in which distance from shoreline was indicated. We verified the distance (*X*) from a shoreline to the entrance of a burrow by comparing our direct, on-site measurement with a visual inspection of our photographs of beach surfaces. Herein, we define *X* = 0 m (shoreline) by the tip of current mark when we started our fieldwork (at 15:15 h on July 24, 2017). The low tide on this date was at 12:39 h and the high tide was at 19:14 h (tide data from the Meteorological Agency of Japan).

At the Ishigaki site, we only measured the horizontal position (*X*) of burrows (14:00 h on May 14, 2016). In this survey, we sampled 23 burrows. The high and low tides at Ishigaki Island on this date were at 13:05 h (low) and 19:55 h (high) (data from the Meteorological Agency of Japan).

### Beach substrate conditions

We made detailed measurement of substrate conditions of the beach at the Tsu site. We measured the vertical slope of beach at every 2 m along transect *X* (Fig 2(f)). We collected 10 samples of beach sand along transect *X*, (at 5, 10, 12.5, 15, 17.5, 20, 22.5, 25, 27.5, and 30 m) by scooping a fixed volume of sand (100 cm^3^) at each transect location. We measured distance from shoreline with a tape measure. We also collected beach sand samples from cores at 10 cm intervals (at depth, *Z*-direction) at three points along each transect (at *X* = 15, 20, and 25 m). We measured groundwater elevation by digging until we reached a stable water level, which we assumed was ground water elevation. We performed these measurements very quickly to minimize the effect of rising tides. We measured water content (*W*) and packing fraction *φ* in all beach sand samples (collected in the Tsu site at both in *X* and *Z* directions) using the following procedures. First, we determined total mass (*M*_total_) of each 100 cm^3^ (*V*_total_) sample of beach sand. Then, we dried the samples and measured the mass of dried sand (*M*_grain_) of each sample. We calculated water mass (*M*_water_) for each sample as *M*_water_ = *M*_total_ − *M*_grain_. We estimated the volume of sand grains (*V*_grain_) and volume of water *V*_water_), *V*_grain_ = *M*_grain_/*ρ*_grain_ and *V*_water_ = *M*_water_/*ρ*_water_, where *ρ*_grain_ = 2500 kg m^−3^ (approximate density of sand) and *ρ*_water_ = 1000 kg m^−3^ (density of water). These values are estimates; actual densities could have been slightly different. We calculated *W* and *ϕ* as,

$$\begin{array}{c} W=\frac{V_{\text{water}}}{V_{\text{total}}}, \end{array}$$ (1)

and

$$\begin{array}{c} \phi =\frac{V_{\text{grain}}}{V_{\text{total}}}. \end{array}$$ (2)

We also measured the grain size distribution (when dried) of sand collected at the Tsu site.

At the Ishigaki site, we only measured water content of sand layers (*W*) at 0, 2, 4, 6, and 8 m from shoreline (*X*) to examine the relationship between beach substrate characteristics and burrow distribution.

We performed laboratory experiments (described below) to ascertain the physical implications of our substrate measurements.

## Experimental methods

We performed a set of experiments to characterize the mechanical properties of wet granular matter by constructing a microcosm with an artificial tunnel structure, in which we used beach sand to encase the tunnels. In similar experiments, we had used spherical glass beads to focus on the intrinsic physical attributes of wet granular matter [8, 22]. In contrast, we conducted this experiment with natural sand to compare the experimental results directly with naturally constructed ghost crab burrows. By comparing experimental results with the field data, we can determine limitations on the mechanical stability of crab burrows.

### Range of water content to maintain a stable tunnel in wet granular substrate

**Fig 3:**
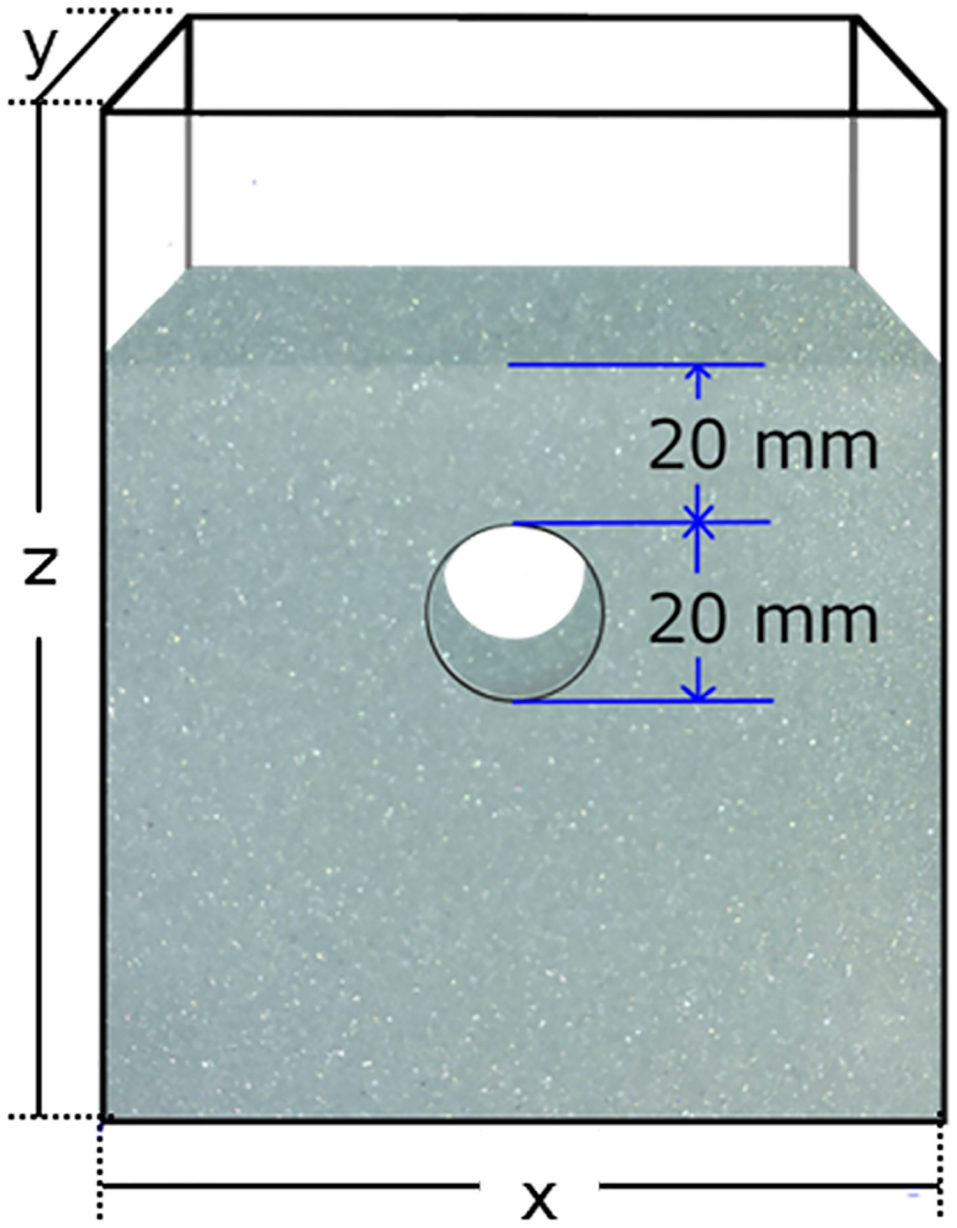
Schematic of an experimental container used to determine stability of a horizontal tunnel through wet granular substrate. This horizontal tunnel was 20 mm in diameter with a 20 mm of overlaying substrate. This container had the following dimensions: *x* = 100 mm, *y* = 50 mm, and *z* = 120 mm, but container sizes and tunnel diameters varied slightly to test different effects of sands on various tunnel diameters.

We investigated the static stability of tunnels in a sand matrix in a microcosm in an acrylic container [100 mm (*x*-direction) × 50 mm (*y*-direction) × 120 mm (*z*-direction)] (Fig 3). We prepared the wet granular matter by mixing grains with tap water in a mixing bottle. Then, we rotated the mixing bottle on a pot-mill rotation table (Nitto Kagaku, ANZ) at 100 rpm for 10 minutes. We placed a solid cylinder (20 mm diameter) horizontally in each acrylic container before pouring the wet granular matter into the container. We created a horizontal tunnel (diameter 20 mm) in the sand substrate by withdrawing the cylinder (diameter *D*_0_) through a hole in the side of the acrylic container, leaving behind the wet sand surrounding the tunnel formed by the cylinder. We examined the static stability of the created tunnels immediately after withdrawing the cylinder because unstable tunnels collapse instantly. Therefore, relatively short-term observations (about 10 s) were sufficient to judge a tunnel’s stability. We replicated each experiment three times to evaluate the precision of our results. We compared tunnel stability with two substrate materials: glass beads (grain diameter *d* = 0.4 mm, true density *ρ*_grain_ = 2500 kg m^−3^) and sand grains collected at the Tsu site that had been sifted (grain diameter *d* < 0.3 mm, true density *ρ*_grain_ = 2500 kg m^−3^). The main control parameter that varied in these experiments was water content *W* (Eq (1)). Cross-sectional diameter of the tunnel (*D*_0_) was held constant at 20 mm for all experiments. Thickness of the granular layer above the tunnel was also held constant at 20 mm (Fig 3). In all runs of the experiment, the packing fraction was controlled at *ϕ* = 0.52.

### Loading compression of the tunnel structure

#### Experimental procedure

We investigated the deformation of the tunnel by applying an external load to the surface of the substrate. The experimental design and procedures were basically identical to those in one of our previous studies [8, 22]. In that experiment (identical setup as Fig 3), the wet granular layer (glass beads) was compressed uniformly at the surface of the substrate (from the top) with a constant speed (0.5 mm s^−1^). This compression rate was slow enough that the neglect of inertia effect during compression could be disregarded. In the current experiment, we varied the following parameters: initial water content *W*_0_, initial packing fraction *ϕ*_0_, and initial cross-sectional diameter of the tunnel *D*_0_. To check the precision of measurements, we conducted each experiment three times under the same initial conditions. We performed experiments over a range of *W*_0_ values from 0.025 to 0.30 and *ϕ*_0_ values from 0.49 to 0.52. All variations of the experiments were similar, but we varied container sizes somewhat so that we could vary the initial diameter of tunnels (Table 1). We used two grain sizes in this experiment, obtained from sand collected at the Tsu site (sifted grain size *d* ≤ 0.3 mm, true density *ρ*_grain_ = 2500 kg m^−3^) and sand collected at the Ishigaki site (sifted grain size 0.3 ≤ *d* < 0.71 mm, true density *ρ*_grain_ = 24500 kg m^−3^). We used natural sand grains to mimic natural substrate conditions through which ghost crabs burrow. The thickness of granular substrate above the tunnels was initially 20.6±2.0 mm in all experiments. Parameter ranges are summarized in Table 2.

**Table 1:** Size of experimental containers (microcosms).

Initial tunnel diameter *D*_0_	*x*	*y*	*z*
10 mm	80 mm	40 mm	105 mm
20 mm	100 mm	50 mm	120 mm
40 mm	80 mm	40 mm	135 mm
80 mm	80 mm	40 mm	175 mm

**Table 2:** Experimental parameters.

Experimental parameter	Parameter range
Liquid content	0.025 ≤ *W*_0_ ≤ 0.30
Packing fraction	0.486 ≤ *ϕ* ≤ 0.505
Initial diameter	*D*_0_ = 5, 10, 20, 40, 80 mm
Grain	Sand in Tsu *d* < 0.3 mm Sand in Ishigaki 0.3 mm ≤ *d* < 0.71 mm

When we compressed the substrate overlying the tunnels, we videotaped the deformation of tunnel cross-sections using a Sentech CMOS camera [STC-MCCM401U3V (4M Color)] at a rate of two frames per second under transmitted light. The acquired images were binarized and the change in diameter of the tunnel was computed from changes in the area of tunnel images, using ImageJ software. We also measured force *F* as the tunnel deformed and the corresponding displacement of the top loading plate using a Shimadzu AG-X universal testing machine. Details of the experimental method can be found in Shinoda et al. [8, 22].

#### Strength attributes of tunnel stability

To estimate the strength of tunnels to withstand compression of a wet granular substrate (including beach sand), we applied a simple quantitative model to determine shear stress at the top of the tunnel [8, 22, 24]. In this model, the maximum shear stress *τ* is written as

$$\begin{array}{c} \tau =\frac{\sigma_{\text{ex}}+\rho gH_{c}}{2\ln\left(2H_{c}/D+1\right)}, \end{array}$$ (3)

where *σ*_ex_ = *F*/*S* is the stress due to external loading by compression force *F* (measured by the testing machine) onto the area *S*(= *x* × *y*), *H*_*c*_ is thickness of substrate overlying the tunnel (≃20 mm), *ρ* is the bulk density of the wet granular layer, *g* = 9.8 m s^−2^ (magnitude of gravitational acceleration), and *D* is the instantaneous diameter of a cross-section of the tunnel, estimated by our analysis of videotaped images of tunnel deformations. Specifically, we obtained all the quantities on the right side of Eq (3) from our experimental data under ambient conditions. This means that the instantaneous stress can be estimated with our available experimental data.

The details of image analysis and example data can be found in Refs. [8, 22]. Using the stress curves, we define two types of mechanical strength attributes that characterize ghost crab tunnels: effective yield stress and maximum stress [8, 22]. Effective yield stress *τ*_yield_, is defined by the value of *τ* at which the tunnel begins to become significantly deformed. In this study, we defined the beginning of deformation as the instant at which the rate of decrease in cross-sectional area of a tunnel exceeds a 1 mm^2^ s^−1^ threshold. *t* = 0 of time *t* is defined by this instant. We also measured the maximum stress (*τ*_max_), which corresponds to the maximum value of *τ* during the deformation process, as long as it could be reasonably measured. In some experimental runs, *τ* seemed to exceed the limit of what was reasonably measurable. In such situations, it was impossible to measure *τ*_max_. Because the value of *τ*_yield_ is affected mainly by initial conditions, it characterizes the mechanical property of a tunnel in the very early stage of deformation, whereas the *τ*_max_ reflects the effect of compression history because it characterizes the mechanical properties of tunnels in later stages of deformation.

## Results and analyses

### Beach conditions

**Fig 4:**
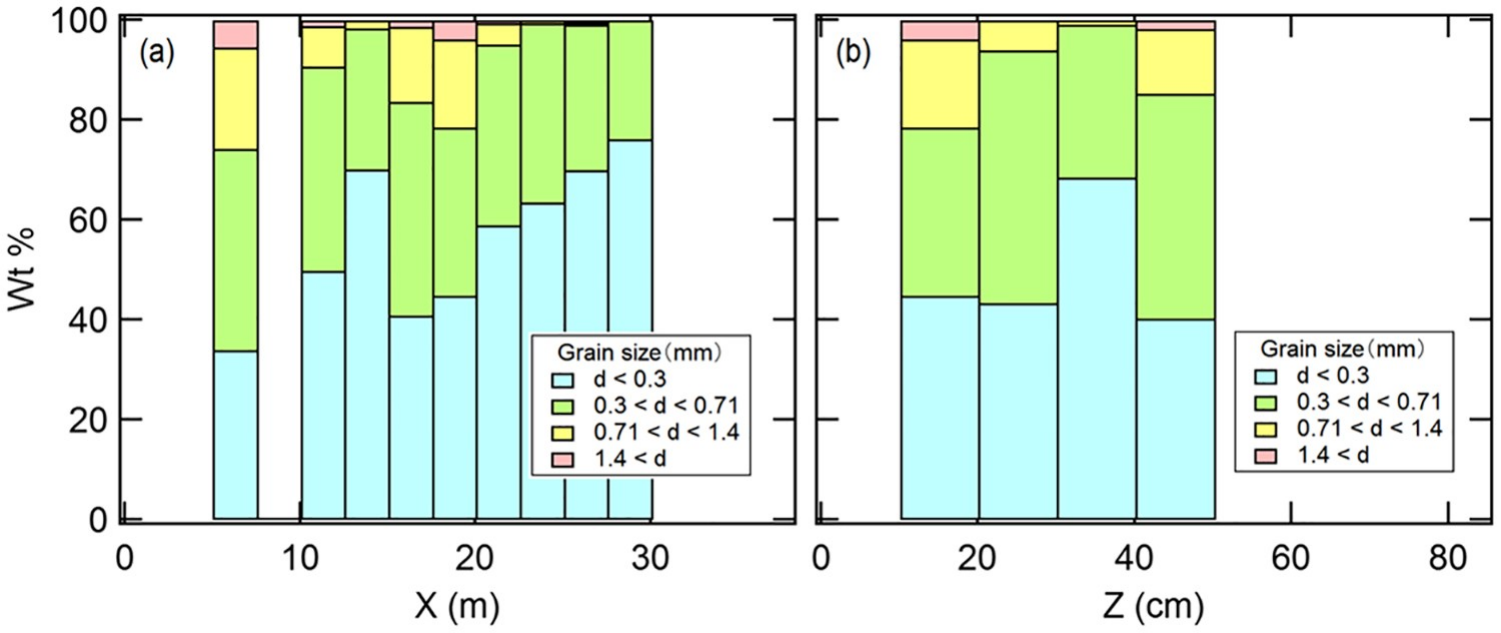
Grain size distribution of beach sand collected at the Tsu site. Panels: (a) The horizontal axis is distance from shoreline (*X*), and (b) is substrate depth (*Z*). Beach sand samples were sifted into four diameter particle sizes: *d* < 0.3 mm (turquoise bars), 0.3 < *d* < 0.71 mm (green bars), 0.71 < *d* < 1.4 mm (yellow bars), and 1.4 mm <*d* (pink bars). *Wt* = percent weight.

Typical grains size at the Tus site was 0.3 mm in diameter (*d*) (Fig 4), which corresponds to the sieve opening for all collected sand samples even though sand size distribution slightly depends on *X* and *Z*. Smaller grains dominate the size distribution in relatively large *X* regime. However, this variation is not significant. Almost all sand samples were dominated by grains with diameters <0.71 mm.

**Fig 5:**
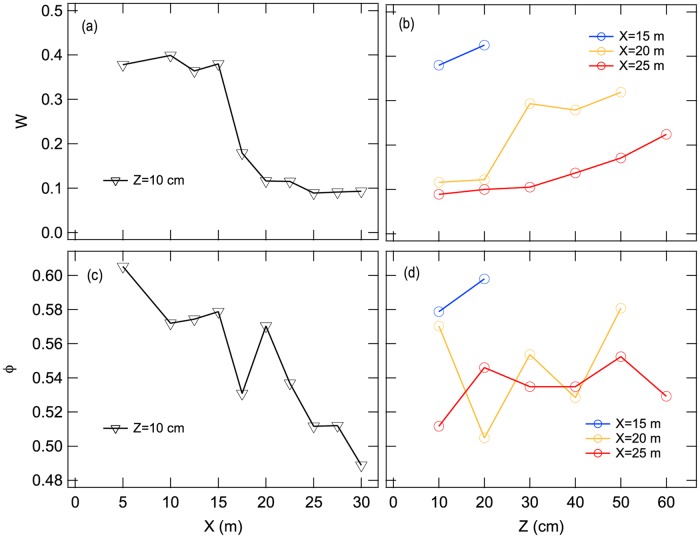
Water content and packing fraction of sand with distance from shoreline and at depth for beach sand sampled at the Tsu site. Panels (a) and (c) depict sample results of water content (*W*) and packing fraction (*ϕ*) at depths (*Z*) 10 cm for each distance from shoreline (*X*). Panels (b) and (d) depict water content (*W*) and packing fraction (*ϕ*) measured at shoreline distances (*X*) of 15, 20, and 25 m.

Measured water content *W* (at *Z* ≃ 5–10 cm) as a function of *X* and *Z* is depicted in Fig 5(a) and 5(b), respectively. As seen in these plots, water content (*W*) depends on both distance from shoreline (*X*) and substrate depth (*Z*). Water content of sands (*W*) drastically declined from about 0.4 to about 0.1 at 17.5 m from the shoreline. Water content also increased with depth. At a depth of 10 cm, water contents at 20 m and 25 m from shoreline were about the same but showed differently higher water content at ≥30 cm depth.

Essentially, *ϕ* declines as a function of distance from shoreline (Fig 5(c)). However, there was no clear correlation between *ϕ* and substrater depth (Fig 5(d)). Raw data to calculate *W* and *ϕ* can be found in S1 File.

### Burrow characteristics

**Fig 6:**
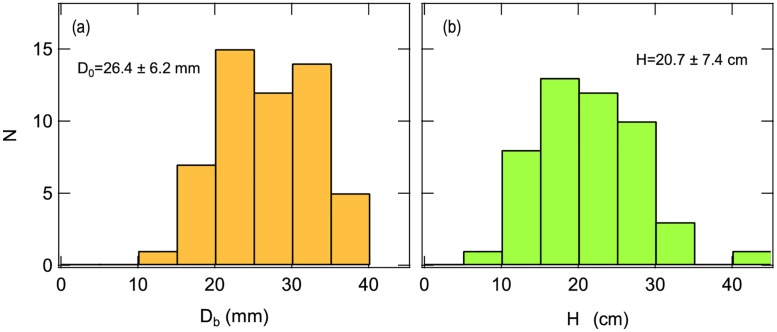
(a) Histogram of burrow diameters (*D*_*b*_) at the Tsu site. The maximum and minimum of tunnel diameter are 38 mm and 10 mm, respectively. (b) Histogram of the vertical depth (*H*) of burrows at the Tsu site. The maximum and minimum of depth values are 43 cm and 10 cm, respectively.

Fig 6 shows the frequency distributions (histograms) of *D*_b_ and *H* measured at the Tsu site. Measured *D*_b_ and *H* data are listed in S2 File. The diameter of burrows (*D*_b_) varied from approximately 10 to 40 mm and the overlying substrate depth (*H*) varied from 5 to 45 cm. The average tunnel diameter was 26.4 mm, whereas average depth was 20.7 cm.

### Relationships between burrow characteristics and beach sand microenvironments

**Fig 7:**
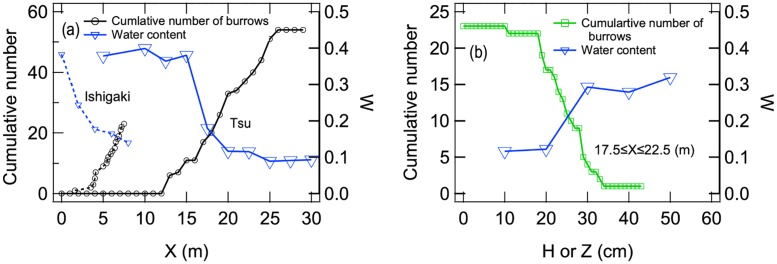
Burrow characteristics at the beach sites. Panels: (a) Cumulative number of burrows whose entrance positions are closer than the depicted distance from the shoreline (*X*) and the water content of sand at distance *X* measured from the shorelines (and the depth *Z* = 10 cm) at the Tsu site (solid curves) and Ishigaki site (dashed curves). (b) Cumulative number of burrows shallower than depth *H* at 17.5–22.5 m distance from shoreline (green squares) and water content of sand versus tunnel depth at 20 m from the shoreline (blue triangles), measured at Tsu site.

We compared the spatial distributions of burrows relative to beach sand conditions (Fig 7). The cumulative number of burrows increased rapidly after about 12 m from shoreline at the Tsu site (approximately when water content declined rapidly from 0.45 to <0.2), whereas the number of burrows increased rapidly after about 4 m at the Ishigaki site (about where water content reached about 0.2) (Fig 7(a)). Sand and burrow data collected at the Ishigaki site are stored in S3 File. Sand water content increased with depth, which negatively affected the depth of burrow tunnels. For example, at the 17.5–22.5 m distance from shoreline, most tunnels were deeper than 20 cm (where water content is about 0.1), but when sand water content increased to 0.3 at >30 cm depth, almost no tunnels extended to that depth (Fig 7(b)). This means that burrows (and their tunnels) are concentrated within a certain zone in both *X* and *Z* directions, based mostly on water content of the sand substrate. Although the distance from shoreline for these zones differed between the two sites we sampled (due to differences in tidal ranges), most burrows are restricted to zones where degree of sand saturation is low (<0.3)].

**Fig 8:**
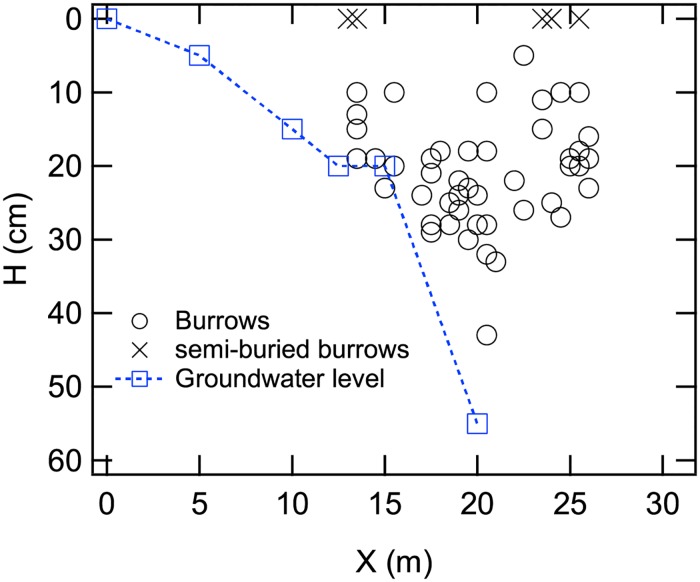
Depth of burrows relative to groundwater elevation and distance from shoreline (Tsu site). Burrows tend to become deeper as distance from shoreline increases. About 15 m landward from shore, the depth of burrows coincides with the groundwater elevation. However, burrows are shallower than the groundwater level at location more than 15 m from the shoreline. Semi-buried burrows (depicted as exes) indicate relatively old (seemingly inactive) burrows that are almost buried.

Almost all ghost crab burrows are concentrated above the water table (Fig 8) because depth to groundwater becomes deeper further from shore. Depth to groundwater levels off abruptly at 20 cm depth about 20 m from shore. However, we did not measure groundwater depth much further from shore (beyond 25 m). Sand at the surface (beyond 25 m) was dry in this zone, and water content at 10 cm depth was about 0.1 (Fig 7(a)). Most of the burrows do not reach the groundwater elevation, except for the burrows located about 15 m from the shoreline (Fig 8).

### Results of the static-stability experiment

**Fig 9:**
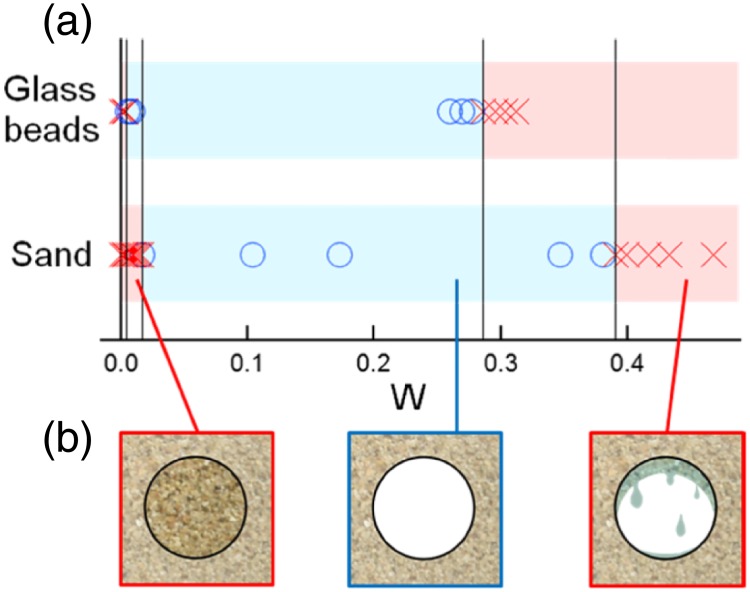
Experimental results on the initial stability of a tunnel in wet granular layer. Panles: (a) Static stability diagram for glass beads and sand. The ranges in water content where a stable tunnel structure can be maintained are indicated by circles and blue color. Unstable regions are indicated by exes and red color. Although a certain amount of liquid is necessary to keep a tunnel stable, too much liquid prevents the formation of a stable tunnel. (b) Three characteristic outcomes are depicted schematically (immediate collapse, stability, and collapse due to exuding water).

The stability-experiment results are presented. As expected, the stability of tunnel structure depends on water content of sand. A phase diagram of tunnel stability (Fig 9) indicates the range of water content (blue region) in which a stable tunnel structure can be maintained. A tunnel structure becomes unstable at extremely small water contents (*W* < 0.003 for glass beads and *W* < 0.017 for beach sand) and at a high water contents (*W* > 0.28 for glass beads and *W* > 0.39 for sand) (Fig 9). In sand with very low water content, tunnel structure collapsed immediately after we extracted the cylinder from the experimental container. Similarly, highly saturated sands exude water that significantly deforms tunnel structures. A stable tunnel structure can be maintained between these two extreme situation limits. This tendency to maintain structure at some interim degree of saturation is common to both glass beads and sand. However, the phase boundaries between stable and unstable states depend on grain type (e.g., glass beads vs. sand). In situations where water content is low, the minimum water content to stabilize a tunnel is larger in sand than in glass beads. In contrast, the upper limit of water content to maintain a stable tunnel is higher for sand than for glass beads.

### Mode of tunnel deformations by compression experiment: Shrink or collapse

**Fig 10:**
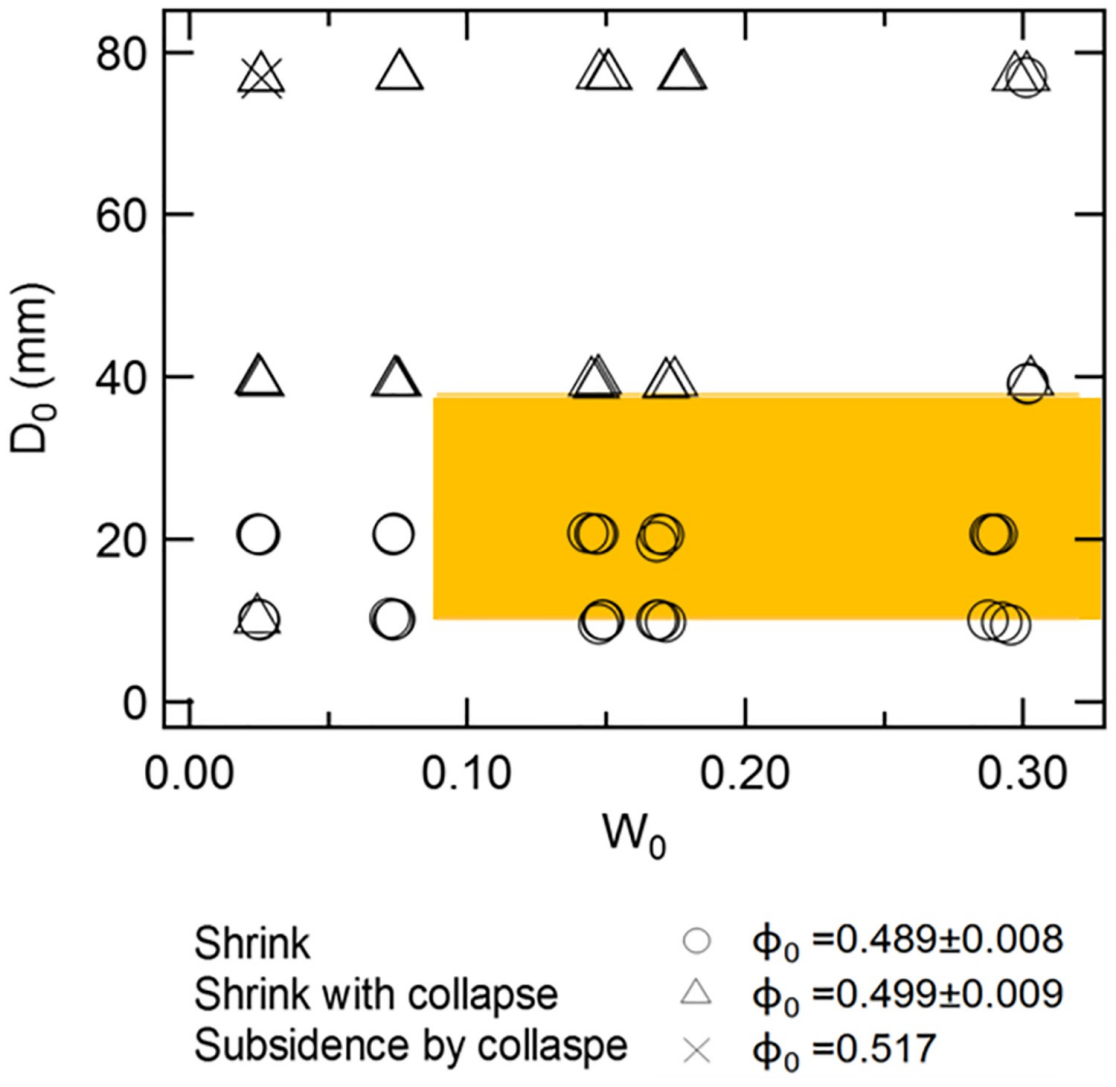
Phase diagram of tunnel deformation/collapse scenarios obtained from experiments with sifted sand collected from the Tsu site (*d* < 0.3 mm). Symbols indicate the three modes of tunnel deformation [shrink (circles), shrink with collapse (triangles), and collapse-by-subsidence (ex)]. The mode of tunnel deformation clearly depends on tunnel diameter (*D*_0_) but is almost independent of water content (*W*_0_) as was the case for glass beads [8]. The collapse-by-subsidence scenario is only observed at a water content (*W*_0_) of 0.02 and tunnel diameter (*D*_0_) of about 80 mm. The range of the actual burrow diameters and corresponding water contents is depicted by the yellow rectangle.

Next, results on the compression experiments are described below. We observed three types of tunnel deformation scenarios in sand: (1) shrink, (2) shrink with collapse, and (3) collapse via subsidence, just as we found for glass beads in a previous study [8]. These three types of collapse scenarios could be a universal attribute of wet granular substrates, independent of grain shape or size. The phase diagram shown in Fig 10 is qualitatively identical to the diagram for glass beads. In Fig 10, circular symbols represent the shrink phase while triangular and cross marks indicate the collapse phases. An initially large diameter tunnel becomes unstable due effects of large voids [8]. However, water content and packing fraction are almost immaterial to mode of deformation. In the case of shrink deformation, a tunnel gradually becomes narrow without any collapse occurring (i.e., tunnel shrinks almost uniformly along the *y*-axis). Therefore, we consider the deformation to be a quasi two-dimensional deformation. Under the shrink with collapse scenario, although the tunnel shrinks continuously, the ceiling of tunnel collapses several times. Collapses often occur after only a little shrinkage. In the collapse-by-subsidence scenario, the upper part of a tunnel completely collapses after only a tiny bit of loading force. A detailed description of these collapse scenarios (with example data using glass beds) are discussed in [8], but we found that the identical scenarios occur in sand. In all these collapse scenarios (regardless of substrate type), the degree of roundness of the cross-section of a tunnel is gradually obliterated by shrinking or collapsing.

### Stress and strengths estimated by the compression experiment

**Fig 11:**
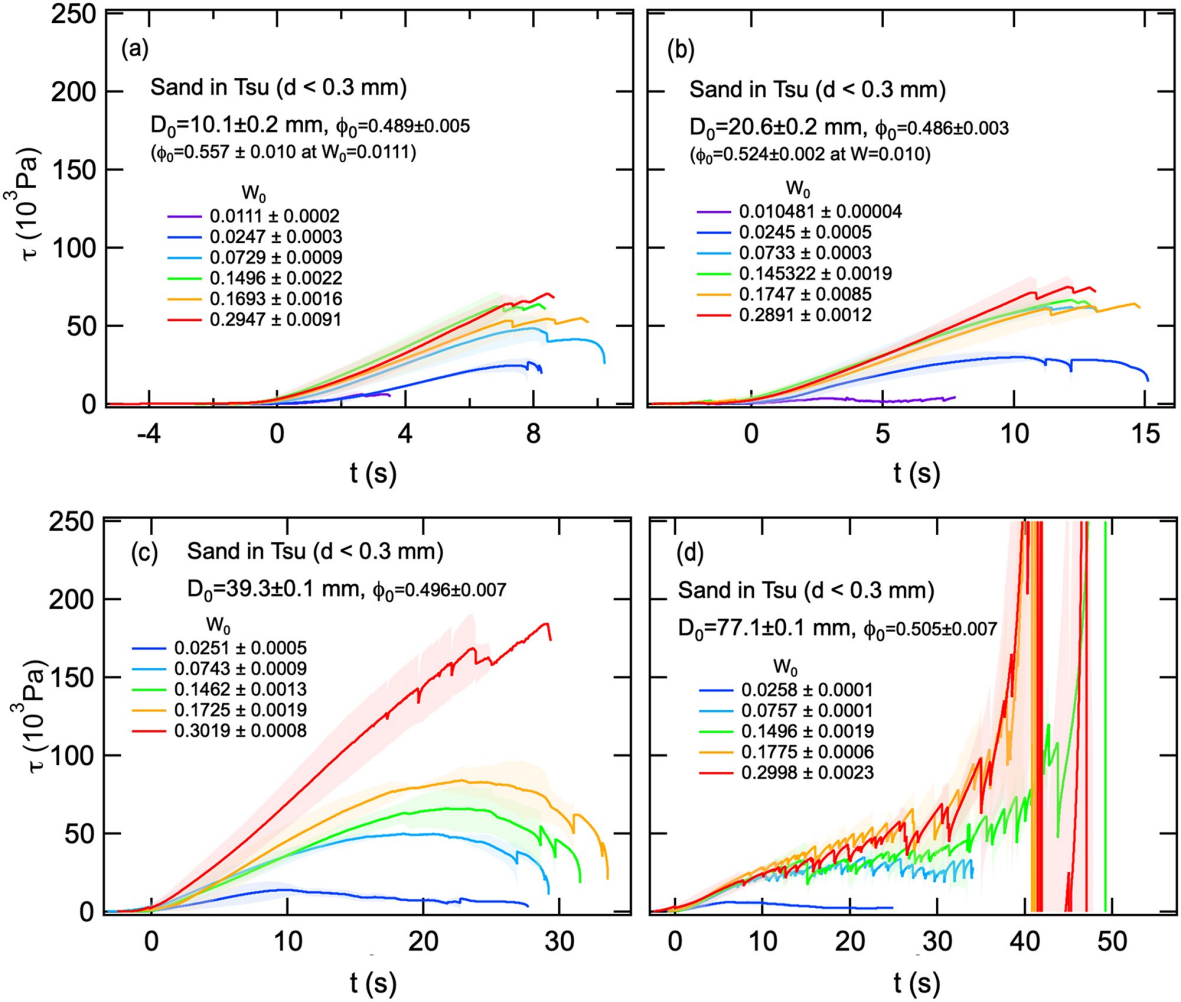
Temporal variations of *τ* obtained by compression experiments with sand collected at the Tsu site. Sand grain diameter (*d*) was < 0.3 mm. The mean (solid curves) and standard deviation (thin bands of matching color) are depicted for three experimental conditions with four tunnel diameters (*D*_0_). Stress *τ*(*t*) curves are depicted for sands with five different water contents (*W*_0_). Panels: (a) *D*_0_ = 10.1 ± 0.2 mm, (b) *D*_0_ = 20.6 ± 0.2 mm, (c) *D*_0_ = 39.3 ± 0.1 mm, and (d) *D*_0_ = 77.1 ± 0.1 mm.

The pressure required to cause tunnel collapse in wet sand depends on the diameter of the tunnel and degree of saturation of surrounding sand. We conducted a compression experiment to tease out this relationship using sand from the Tsu site (*d* < 0.3 mm) with four tunnel diameters (10–77 mm) and water content (0.01–0.3) (Fig 11). We found that when tunnel diameters were small (*D*_0_ ≤ 20 mm), stress curves are essentially smooth (Fig 11(a) and 11(b)). The sudden spurts observed in the late stage of the stress curves in Fig 11(a) and 11(b) are due to averaging three experimental runs. Tunnels with intermediate diameters (*D*_0_ = 39 mm) show relatively small-scale instability before collapse (Fig 11(c)), but such collapses are almost averaged out by averaging three experimental runs. Large diameter tunnels (*D*_0_ = 77 mm) show spurts throughout the stress curve due to high instability, a response of collapse (Fig 11(d)).

**Fig 12:**
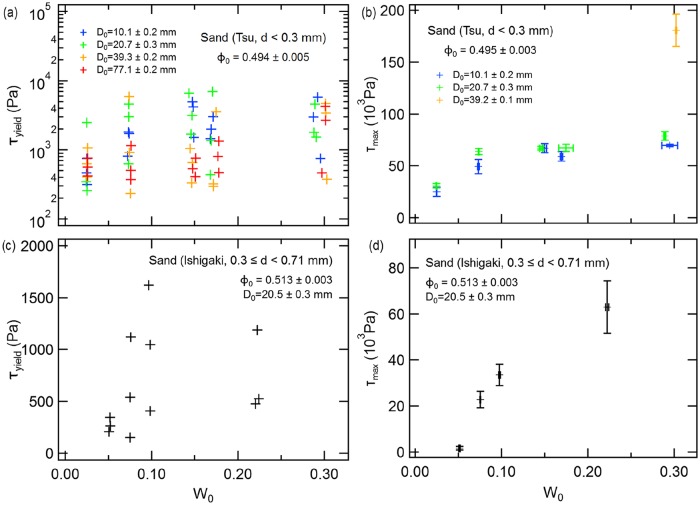
Effective yield stress (*τ*_yield_) and maximum stress (*τ*_max_) in wet sand of two diameters (from the Tsu and Ishigaki sites) relative to various tunnel diameters subjected to compression of overlying sand substrate. Panels: (a) Water content (*W*_0_) relationship between effective yield stress (*τ*_yield_) using sand collected at the Tsu site (*d* < 0.3 mm) relative to various tunnel diameters *D*_0_, (b) water content (*W*_0_) relationship between maximum stress (*τ*_max_) using sand collected at the Tsu site relative to various tunnel diameters (*D*_0_), (c) water content (*W*_0_) relationship between effective yield stress (*τ*_yield_) using sand collected at the Ishigaki site (sand diameter 0.3 < *d* < 0.7 mm) relative to tunnel diameter (*D*_0_) of 20.5±0.3 mm, and (d) water content (*W*_0_) relationship between maximum stress (*τ*_max_) using sand collected at the Ishigaki site.

When we tested resistance to compression of wet sands collected from our two field sites, we found that effective yield (at *τ*_yield_) typically occurred at about 10^3^ Pa, whereas maximum stress (*τ*_max_) typically ranged around 10^4^–10^5^ Pa. We could not discern a clear relationship between water content and effective yield strength (Fig 12(a) and 12(c)). That is, effective yield strength is almost independent of water content. In contrast, maximum stress monotonically increases with increasing water content (Fig 12(b) and 12(d)). Experimental conditions and measured *τ*_yield_ and *τ*_max_ values are listed in S4 File.

### Size and spatial restrictions on burrows

Combining insights gleaned from our experimental results and field observations, we investigated physical restrictions on the practical size of crab burrows by considering the mechanics involved with determining the potential range in diameter of crab burrows relative to the tunnel deformation scenarios we examined. The diameter of crab tunnels on the beaches we studied ranged from 10 to 40 mm (Fig 6). The water content of beach sand where burrows were located ranged from approximately 0.1 to 0.4 (Fig 5 and yellow rectangle in Fig 10). Thus we can infer that the yellow rectangle in Fig 10 represents the regime under which continuous shrink deformation occurs. Therefore, we confirm that burrows are constructed under conditions that prevent (or resist) collapse (in balance with tunnel diameter, sand grain size, and sand water content). When loading is exerted on the layer above small burrows, continuous shrinkage would occur, rather than a sudden collapse.

When we experimentally analyzed the relationship between the spatial distribution of burrows and water content of beach sand through which crab tunnels are built, we found that burrows could be constructed in sands within a relatively wide range in water content (0.017 ≤ *W* ≤ 0.39) (Fig 9). Indeed, this is what we found in the field (Fig 7). Because the phase boundary between shrink and collapse phases does not clearly depend on water content (Fig 10), water content is not a very important regulating factor as long as a stable tunnel can be constructed. Therefore, crabs can construct burrows in sands that possess a relatively wide range of water content (Fig 7), which is advantageous in that tidal fluctuations and precipitation events act to change water content of beach sands over time. In the real world, the spatial distribution of burrows (based on stability) is constrained almost only by the water content (saturated conditions) through which tunnel structure are built.

### Stress-based restrictions on vertical depth of burrows

Most ghost crab burrows are less than 30 cm deep (Figs 6(b) and 7(b)). This depth is shallower than typical groundwater depths where crabs build their tunnels (Fig 8). In addition, water content of beach sands is within the range that allows crabs to build a stable tunnel at any depth. Thus, water content is probably not a major factor constraining the depth of tunnels.

**Fig 13:**
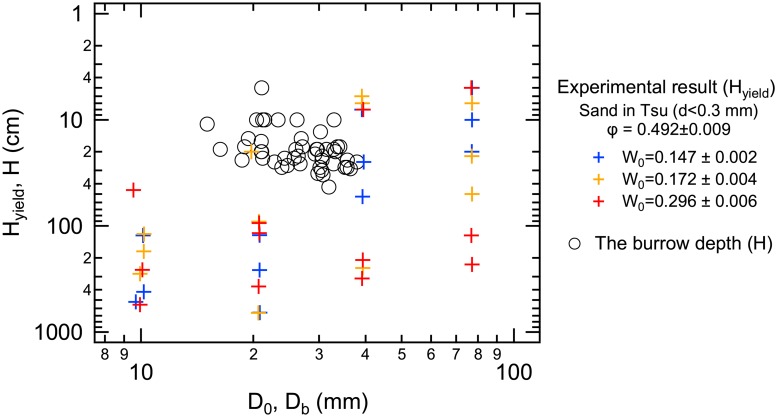
Relationship between tunnel diameter and depth of actual burrows (circles) and an experimentally obtained pressure yielding depth *H*_yield_ (plus marks). Tunnel diameter (*D*_0_) relationship with *H*_yield_ was computed from experiments (plus marks) with sand collected at the Tsu site of grain diameter *d* < 0.3 mm. Colors differentiate various water contents in sand (*W*_0_). Circular symbols indicate the relationship between the thickness of sand overburden (*H*) and tunnel diameter (*D*_*b*_) of actual crab burrows observed in the field.

It is important to determine what the maximum thickness of an overlying wet sand layer would be that would not cause the deformation of tunnels (i.e., without any external pressure applied from above), which we designate herein as *H*_yield_. In other words, *H*_yield_ indicates the depth at which a crab tunnel (especially the lower chamber) would be deformed by gravitational pressure exerted by an overlying burden of wet sand. *H*_yield_ is calculated by using Eq (3) with *σ*_ex_ = 0 and *τ* = *τ*_yield_. The experimentally obtained *H*_yield_ and actual depth of crab burrows (*H*) measured in the field works are plotted in Fig 13. The plus symbols indicate the experimentally obtained yield depth *H*_yield_, and the circular symbols represent the actual crab-burrow depths *H*. Although there are some overlaps between *H*_yield_ and *H*, the two parameters mostly separated on the graph. Most crab burrows are shallower than the experimentally estimated yield depth (*H*_yield_), implying that the natural crab tunnels are sufficiently rigid to prevent them from deforming in response to gravitational pressures of overlying sand.

## Discussion

### Burrow dimensions

One previous study [10] showed that the tunnel diameters correspond to the width of a crab’s carapace. That is, larger (older) crabs create wider burrows than smaller (younger) crabs. Although in our study, we did not measure the size distribution of ghost crabs populations on the beaches we studied, we did determine average values for both burrow entrance diameters (*D*_b_) and burrow depth (*H*) that satisfy the stable conditions for tunnels. This result is natural since ghost crabs would have their typical carapace size.

The dimensions (diameter and depth) of crab burrows are small enough to prevent collapse due to pressures exerted by the sand overburden. Because we compared the gravitational weight of sand overburden (*H*_yield_) with the lower limit of applied stress that would cause a small deformation, *H*_yield_ is rather a strict criterion to assess the risk of tunnel deformation (collapse). Nevertheless, sand crabs maintain their tunnels within this strict criterion. In addition, the diameters of crab tunnels are small enough to resist a sudden collapse of their tunnels. In fact, our mechanical approach could be used to estimate the practical upper limit for a tunnel diameter (and hence, crab size) on a sandy beach.

### Stability and strengths of a tunnel in wet sand

The angular shape, surface roughness, and size of sand grains are possible reasons that critical water content required to stabilize crab tunnels varies among beaches. The complex structural interactions of an interstitial liquid in wet granular matter must be properly considered when predicting tunnel stability under varying conditions. The effect of interstitial water in sand varies in response to the variety of water contents among sand grains of variable shapes and sizes [15]. If water content is low, water bridges form between grains, which act as a cement binding grains together, which in turn enables a tunnel to maintain its stability. The lower limit of water content that allows the formation of water bridges is higher for rough grains than spherical grains [25]. In general, a wetting liquid is trapped better on rough surfaces [25, 26]. Therefore, the lower limit of water content for supporting a stable tunnel would be larger for rough grained sands than smooth sands. Our experimental results were qualitatively consistent with this prediction. Furthermore, as water content increases, the distribution of water across sand grains becomes more continuous. Eventually, with more interstitial water, friction among grains is reduced (due to the lubricating effect) so much so that a tunnel cannot maintain its stability and then collapses. That is, under highly saturated conditions, interstitial liquid (e.g., water) enables a granular layer (e.g., sand) to flow as the grains slip past one another. This lubricating effect is enhanced in smooth and spherical particles. That is the reason why the upper limit of water content required to maintain stability with glass beads is less than with sand. In other words, the range in water content to maintain stability is wider in sand than in glass beads (i.e., natural, wet sand of beaches is more stable than artificial, uniformly-shaped and sized wet spheres).

Typical values for effective yield stress (*τ*_yield_) in sand is about 10^3^ Pa, whereas maximum stress (*τ*_max_) ranges from about 10^4^ to 10^5^ Pa These values are about 1–2 orders of magnitude higher than for wet glass beads [8, 22]. The principal reason for this difference is the rough and angular shape of sand grains. Such irregular shapes provide stronger connections (friction) among grains. Thus, a larger force is required to compress a granular layer composed of natural sand grains than is required for glass beads. The large fluctuation in measured strength implies that grain shape and large variation in shape, size, and roughness among grains definitely affects the strength of tunnel structures.

## Conclusions and future questions

Ghost crab burrow tunnels vary within a narrow range of diameters and depths even though the water content of the beach sands through which they burrow varies widely in space. The spatial distribution of crab burrows is related to the distribution in water content of beach sands. Based on our lab experiments, we identified how water content in sands is related to tunnel stability. We also found that tunnels are deformed in a variety of ways, mainly dependent on the initial diameter of tunnels. We defined two types of tunnel strengths, based on effective yield and maximum strengths, which relate to the effective and maximum force that can be applied to sand overlying a tunnel when it deforms by the overburden weight.

We found that the typical diameter of crab burrows is small enough to prevent a sudden collapse. We revealed that the range in water content, which is spatially distribution with distance from shoreline (due to tidal influences), restricts where crab tunnels will be stable and thus restricts where crab tunnels occur on a beach (i.e., distance from shoreline). However, the depth of burrows on beaches cannot be simply predicted by water content. Rather, we revealed that the burrows are built shallow enough to avoid even small tunnel deformations from pressure exerted (via gravity) by sand layer overlying the burrows.

As mentioned in the introduction, the stability of void structures in cohesive granular matter can be applied to a variety of scientific and engineering problems. Although this study focuses on the stability of sand crab burrows, the results we obtained on tunnel strength could provide insights into behaviors of various other void-containing structures.

To fully understand the physics of crab burrows, it will be necessary to comprehensively evaluate other factors not considered in this study, for example, the effects of temperature and humidity on sand crabs, moisture-dependent resistance of sand layers against tunneling, the burrowing behaviors of sand crabs, and temporal variations in the surrounding environment. Geological setting should also be examined more in detail. Furthermore, we have not considered the temporal variation of tidal effects that might submerge or inundate burrows or whether crabs move their tunnels landward in response to tidal variations, which would require crabs to construct new burrows. Although we neglected temporal variations in the construction of burrows, we determined that ghost crabs construct their tunnels in zones that are appropriately moist, and that their tunnel dimensions (and crab size) are small enough to prevent deformation and collapse of their tunnels. This study provides an important step in quantitatively examining the physics underlying the structure of crab burrows in beach sand.

## Supporting information

S1 File Raw data of wet sand collected at the Tsu site. (CSV)

S2 File Raw data of burrow size (diameter *D*_b_ and depth *H*) measured at the Tsu site. (CSV)

S3 File Sand and burrow data collected at the Ishigaki site. (CSV)

S4 File Table of experimental conditions and measured *τ*_yield_ and *τ*_max_. (CSV)
